# Partial differential equation models for invasive species spread in the presence of spatial heterogeneity

**DOI:** 10.1371/journal.pone.0300968

**Published:** 2024-04-02

**Authors:** Elliott H. Hughes, Miguel Moyers-Gonzalez, Rua Murray, Phillip L. Wilson

**Affiliations:** 1 School of Mathematics and Statistics, University of Canterbury, Christchurch, New Zealand; 2 Mathematical Institute, University of Oxford, Oxford, United Kingdom; 3 Te Pūnaha Matatini Centre of Research Excellence, University of Auckland, Auckland, New Zealand; Center for Advanced Systems Understanding (CASUS), GERMANY

## Abstract

Models of invasive species spread often assume that landscapes are spatially homogeneous; thus simplifying analysis but potentially reducing accuracy. We extend a recently developed partial differential equation model for invasive conifer spread to account for spatial heterogeneity in parameter values and introduce a method to obtain key outputs (e.g. spread rates) from computational simulations. Simulations produce patterns of spatial spread which appear qualitatively similar to observed patterns in grassland ecosystems invaded by exotic conifers, validating our spatially explicit strategy. We find that incorporating spatial variation in different parameters does not significantly affect the evolution of invasions (which are characterised by a long quiescent period followed by rapid evolution towards to a constant rate of invasion) but that distributional assumptions can have a significant impact on the spread rate of invasions. Our work demonstrates that spatial variation in site-suitability or other parameters can have a significant impact on invasions and must be considered when designing models of invasive species spread.

## 1 Introduction

Invasive species harm biodiversity, damage agriculture, and can even increase the risk of wildfires or other natural disasters [1]. A wide variety of different species can pose risks; from pine trees to feral cats to microscopic viruses [1]. The damage caused by individual invasive species can vary dramatically, but in some cases multiple species extinctions or extirpations can be traced back to the introduction of a particular organism. For example, the introduction of the Brown Tree Snake *Boiga irregularis* to the island of Guam led to the “nearly complete extirpation of native bird species” [2, p. 46]. While total costs of all invasive species globally are understudied and hard to estimate [3, 4], the economic impact of invasive species globally was estimated to exceed $423 billion (2023 American dollars) in 2019 [5]. Because of these costs, understanding the processes that underpin invasions is an important goal of ecological research.

Information on how a biological invasion may evolve over time allows managers and conservation practitioners to better allocate resources, prioritise responses to multiple threats, and develop strategies to prevent or mitigate the impacts of an invasion. On a societal level, understanding the evolution of a biological invasion increases accuracy of estimates of the risks posed by invasions and the costs of inaction.

Mathematical modelling is frequently employed in ecological research [6, 7]. Models of ecological processes have been used to understand the stability of ecosystems to perturbations [7], complex dynamics in the reproductive biology of parasites [8], and (with particular relevance to this work) to understand and predict the spread of invasive species [6]. Such models of invasive species spread can provide valuable insights to managers [6] and these insights have been employed to successfully manage biological invasions [9]. For example in [9] models were employed to simulate the response of Coypus (an invasive rodent) populations to climactic variation and management effort, enabling a successful eradication program to be implemented.

Models for invasive species spread can be continuous (e.g. [10, 11]) or discrete in time (e.g. [12–14]) and often feature a spatial component. Such explicitly spatial models are particularly valuable in cases where invasive species are still spreading throughout a landscape (or network of landscapes) and thus the size of the invaded region may be a particularly important quantity of interest to management specialists. Spatial models can also simulate the consequences of management strategies which vary in space. Since actual invasions are often complex phenomena with multiple source populations or invading fronts, understanding how different spatial allocations of effort affect long-term dynamics can be an important goal of ecological models [15].

Common spatially explicit approaches to modelling invasive species spread utilise partial differential equations (PDEs) or integrodifference equations [6]. PDE models have been utilised since the mid -twentieth century to model invasive processes [10] and can be extended to incorporate the interactions between multiple species [16]. Integrodifference models, meanwhile, can be easily modified to incorporate arbitrary dispersal probability kernels [17] (extensions to multiple species/lifestages also exist, see for example [18]). Such flexibility is particularly valuable for species where the spread process is wind-dispersed or otherwise heavy tailed [17, 19]. Integrodifference matrix models (IDMs) attempt to synthesize the advantages of both models, although the theory of such models is not complete [12].

Furthermore, even spatially explicit models of invasion often ignore within-landscape spatial heterogeneity (e.g. local changes in site-suitability). There is some evidence that this approach may be appropriate in certain cases [20] and imposing homogeneity across a landscape significantly simplifies analysis, but such simplifications will not be appropriate in all cases.

A number of papers explore the implications of fragmented habitats or climactic change for integrodifference models. For the latter, the resulting temporally evolving habitat suitability is treated as a sliding window of ‘good’ habitat in [21] and as an expanding or contracting region in [22]. Closer to the theme of our paper, [23, 24] both present integrodifference models for spread in heterogeneous environments. In both cases these models simulate dispersal over a 1D domain with a kernel that varies rapidly depending on terrain characteristics, before utilising homogenisation methods to obtain a smooth profile. However, while the 1D assumption may be necessary to retain analytical tractability, the two-dimensional (2D) geometry of actual habitats may have a non-negligible impact on spread. The combination of multiple species (or lifestages) and spatially heterogeneous environments also significantly increases the mathematical complexity of models and some common simplifications may not be suitable (e.g. theory for IDM models in the presence of spatial heterogeneity has yet to be developed).

PDE models of spatial spread have also been modified to incorporate spatial heterogeneity, often utilising homogenisation methods [25–27]. Prior work has extended simple reaction-diffusion models to incorporate small scale variation in site suitability, which is then ‘averaged out’ to obtain a spatially homogeneous landscape scale model [27]. It is relatively straightforward to incorporate multiple species or lifestages into such models, such as in [27, 28] (for multiple species) or [29] (for multiple lifestages). Much like the integrodifference models discussed above, these studies assumed that the landscape was 1D or that spatial variation only occurred in one dimension.

There are also a number of works utilising cellular automata models to simulate biological invasions in the presence of spatial heterogeneity. We will treat two of these ([15, 30]) in more detail below, as they deal with a similar study system to the model we extend in this paper. Other works utilising similar methods include [31, 32], among others. Such cellular automata models are a promising approach to modelling invasions with spatial heterogeneity, although past models often simulate relatively few landscapes (or focus on a particular extant landscape) and thus can be difficult to generalise.

In this paper, we explore computationally the impact of allowing spatial variation in parameters in an existing model for invasive species spread. We find that incorporating spatial heterogeneity in parameters can have a significant impact on the qualitative appearance of the solution. In particular, we find that incorporating spatial heterogeneity results in solutions which appear qualitatively similar to observed invasions. We also observe that some features of solutions are conserved under spatial heterogeneity (such as overall structure of invasions as almost static for a significant period of time before a rapid evolution towards a constant rate of advance). Our work builds on prior models exploring spatial spread, but considers a wider range of landscape geometries than previous works.

Our results emphasise the importance of spatial structure in mediating invasions. We find that both the existence of variation in parameters and the distribution of parameters in space can significantly impact observed dynamics. Whether spatial heterogeneity accelerates or retards invasions depends on the distribution of parameters and the period of time considered.

As an example system, we consider invasions of grassland ecosystems by exotic conifers. Such invasions are well documented in New Zealand, South Africa, and Chile (among other Southern Hemisphere countries) [33]. The most common invasive species are trees from the family *Pinaceae*, although other species sometimes also pose risks [34]. Because these species are often important economically as forestry trees, managers and conservation specialists need information to contain invasions and assess the risk of new plantings [34]. Consequently, there is a moderately sized literature mathematically modelling the spread of exotic conifers (sometimes informally known as wilding pines). For a review of these models, see [35] or [36]. However, most of these models ignore spatial variation in parameters, except [15, 30].

In both [15, 30], the authors incorporate spatial heterogeneity into a discrete-space, discrete-time, cellular automata model for pine spread. A given domain (8km × 8km in [15]) is divided into square cells of equal size (20m × 20m in [15]) and pines are ‘seeded’ onto a small subset of these cells. Pine spread is managed by semi-deterministic rules (e.g. seeds from a tree are spread into cells according to some probability distribution but growth once established is deterministic). Habitat suitability is either generated from existing maps of a particular landscape [30] or generated using a spatially autocorrelated random process. In both cases one or more parameters are scaled by a fixed amount in the ‘bad’ habitat to reduce the establishment rate, fecundity or survival rate of trees in these less supportive environments.

In [15] the extent of this bad habitat was shown to affect the proportion of the cells invaded, at least at the level of the management unit (e.g. 2km × 2km subregions used to determine the allocation of management resources) but [15] do not claim to find an effect at the regional levels. However, it is important to note that [15] base this assessment on the relative importance of parameters, not their absolute importance. Since this analysis is also based on simulated invasions across only three different spatial configurations, it is difficult to have high confidence in these predictions. In the case of [30], only one spatial configuration is considered so it is not possible to draw inferences on the impact of spatial heterogeneity on model results.

## 2 Methods

To explore the impact of spatial heterogeneity on invasions we employed the model of [36] (see also [35, 37]). This partial differential equation (PDE) model is well-suited to exploring spatial heterogeneity as it is spatially explicit and invasions can be directly simulated, unlike some IDM models [36]. Furthermore, unlike the cellular automata models discussed above, this model allows growth rates to depend on the total amount of biomass present in a location (rather than keeping growth rates constant until the carrying capacity is reached).

In [36], a 1D version of this model is introduced to model the invasion of invasive pines from a shelterbelt or other isolated point source. While the interested reader is encouraged to consult [35] or [36] for details, for convenience a brief summary is presented below.

The model tracks the evolution of ‘adult’ biomass *A* (e.g. biomass from reproducing trees) over a landscape of fixed width 2*L*. The production of new seeds and the dispersal process is modelled by the introduction of a potential biomass variable *C*, which is produced by adult biomass, diffuses outwards, and transitions to the adult state at a constant rate. Potential biomass production at a point is modelled by a hill function of *A* (this functional form is chosen so that there is a weak Allee effect, accounting for the fact that smaller populations are likely of younger, less productive trees), while adult biomass growth is modelled by a logistic function. After suitable scalings, this leads to the coupled partial differential equation

$$\begin{array}{c} \frac{\partial A}{\partial t}=\epsilon C+\rho_{0}A\left(1-A\right), \end{array}$$ (1)

$$\begin{array}{c} \frac{\partial C}{\partial t}=D\frac{\partial^{2}C}{\partial x^{2}}-C+\frac{A^{2}}{\beta^{2}+A^{2}}, \end{array}$$ (2)

with *ϵ* ≪ 1 and all other parameters of order one. A heuristic interpretation of the parameters is given in Table 1.

**Table 1:** Heuristic interpretation of the nondimensional parameters in Eq 1.

Name	Interpretation
*ϵ*	Term governing the transition of potential biomass to reproducing trees. The smallness of this parameter accounts for the relative size of difference of just reproducing and fully mature trees.
*ρ*_0_	Term governing the initial growth rate of adult trees.
*D*	Diffusion coefficient governing the spread of potential biomass (e.g. the expansion of locations where trees could appear).

**Fig 1:**
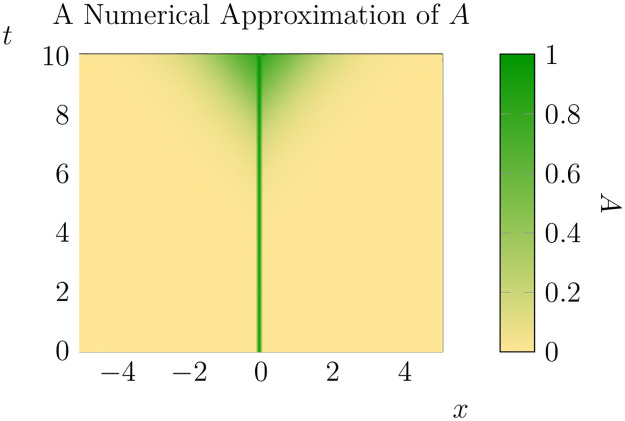
An example of the qualitative dynamics of the 1D model from *t* = 0 to *t* = 10. In dimensional terms, this corresponds to an invasion over a domain approximately 10km square after about 100 years (for a discussion of the dynamics of the invasion process see [36]). Parameter values are as in [36]. Values of *A* near one represent dense forest near carrying capacity, while values near zero represent largely uninvaded areas.

Note that the implied dispersal process has a Laplace kernel, so that this model assumes a dispersal process that has much heavier tails than a standard reaction-diffusion model [35]. In [36] the following initial and boundary conditions are proposed

$$\begin{array}{c} \frac{\partial C}{\partial x}{|}_{-L}=\frac{\partial C}{\partial x}{|}_{-L}=0, \end{array}$$ (3)

$$\begin{array}{c} C(x,0)=0, \end{array}$$ (4)

$$\begin{array}{c} A(x,0)=\{\begin{array}{cc} 1 & x\in [-L/n,L/n]\\ 0 & \text{otherwise} \end{array}, \end{array}$$ (5)

corresponding to a zero-flux requirement across the boundary and an initial biomass distribution which is approximately at carrying capacity in a small strip at the centre of the domain and zero elsewhere. These boundary and initial conditions are chosen as an idealisation of spread from a shelterbelt into a valley, based on the observation that invasive conifer spread in the Southern Hemisphere often occurs from human managed sources (see [14, 38] for prior models of a similar scenario and [33]). In [37] this model is simulated with *ϵ* = 10^−2^, *ρ*_0_ = 1 and *D* = 1, resulting in invasions that are static for a long time period before rapidly evolving towards a constant rate of advance. An example of the numerical output is shown in Fig 1.

Given this 1D model, we first extend it to a 2D domain before allowing parameters to vary in space.

### 2.1 Invasions on a 2D domain

**Fig 2:**
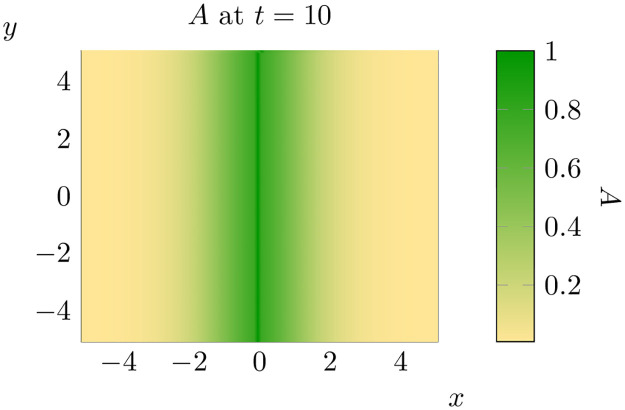
The state of an invasion on a 2D domain at *t* = 10. Dimensional in this and following figures are as in the previous figure.

In a 2D domain, we consider a very simple extension of the model given in [36]

$$\begin{array}{c} \frac{\partial A}{\partial t}=\epsilon C+\rho_{0}A\left(1-A\right), \end{array}$$ (6)

$$\begin{array}{c} \frac{\partial C}{\partial t}=D_{1}\frac{\partial^{2}C}{\partial x^{2}}+D_{2}\frac{\partial^{2}C}{\partial y^{2}}-C+\frac{A^{2}}{\beta^{2}+A^{2}}, \end{array}$$ (7)

where $D_{1},D_{2}\in R$. We will impose that spread is approximately isotropic and that *D*_1_, *D*_2_ are O(1) (in practice a correct choice of scales will guarantee that this is the case). In particular we require that *D*_1_ = *D*_2_ = *D* ∼ 1 and assume other parameters are of the same order as in [36]. For an initial condition we consider (by analogy with a shelterbelt)

$$\begin{array}{c} A(0,x,y)=\{\begin{array}{cc} 1 & x\in [-L/n,L/n],\\ 0 & \text{otherwise}, \end{array} \end{array}$$ (8)

$$\begin{array}{c} C(0,x,y)=0, \end{array}$$ (9)

with Neumann boundary conditions as in [36]. To simulate this model using a finite difference method, we apply a second-order in space (utilising a standard five-point stencil) first-order in time approach and use ghost points to account for the Neumann boundary conditions (see Fig 2). In all cases except Fig 8 (see below for details) we utilised a grid spacing of Δ_*x*_ = 10^−1^ and a timestep of Δ_*t*_ = 10^−3^.

### 2.2. Spatial heterogeneity

With this 2D model in hand, we consider spatial variation in parameter values. There are plausible reasons to expect spatial heterogeneity to either increase or reduce invasion speeds. One possibility, discussed in [39], is that spatial heterogeneity will lead to landscape ‘fragmentation’ with invasions slowed or even halted by locations with very low suitability for the invasive species blocking onwards spread. Alternatively, one could imagine a scenario where the presence of locations with very high suitability led to clusters of high population density, driving rapid invasion of the surrounding less suitable areas. The second possibility is especially salient in the context of pine trees, where we might expect that long distance dispersal could carry seed from high suitability areas over a large portion of the landscape.

As an example of our approach let us first consider a simple extension of our model, allowing initial growth rates (e.g. *ρ*_0_) to vary spatially in a random fashion. This approach models a scenario where site suitability varies in space, but total fecundity and maximum density remains constant. This represents an advantage over models discussed in [39] which do not disentangle the effects of varying site suitability and fecundity at each location.

Since we do not fit the model to real world data, we limit ourselves to computational experiments to explore what dynamics are possible. Our new model now becomes

$$\begin{array}{c} \frac{\partial A}{\partial t}=\epsilon C+\rho_{0}\left(x,y\right)A\left(1-A\right), \end{array}$$ (10)

$$\begin{array}{c} \frac{\partial C}{\partial t}=D\nabla^{2}C-C+\frac{A^{2}}{\beta^{2}+A^{2}}. \end{array}$$ (11)

Where we now define *C* and *A* as functions from $\Omega \times R^{+}\to R$ (where $\Omega \subseteq R^{2}$) and *ρ*_0_ represents a realisation of a random variable. The 2D case was considered here as we expect that considering only a 1D representation of the landscape might artificially increase habitat fragmentation. Furthermore, since structurally a 1D model is equivalent to assuming that spatial variation only occurs in one direction, this 2D approach is generally more realistic.

### 2.3 Generation of *ρ*_0_(*x*, *y*)

We take *ρ*_0_(*x*, *y*) at each point on the grid as samples from a random variable. In particular, for a grid of size *N* × *N*, a grid of size (*N* + 2) × (*N* + 2) of random variates *α*_*i*,*j*_ drawn from a normal distribution with mean one and standard deviation one was generated. Note that these parameter values are arbitrary, but are chosen for simplicity and so that *ρ*_0_ should be positive almost everywhere (after correlating adjacent points—see below). Then, for the point (*x*_*i*_, *y*_*j*_) on the lattice of size *N* × *N* the value of *ρ*_0_(*x*_*i*_, *y*_*j*_) was taken as

$$\begin{array}{c} \rho_{0}\left(x_{i},y_{j}\right)=\frac{1}{9}\sum_{k=i-1}^{i+1}\sum_{l=j-1}^{j+1}\alpha_{k,l}. \end{array}$$ (12)

**Fig 3:**
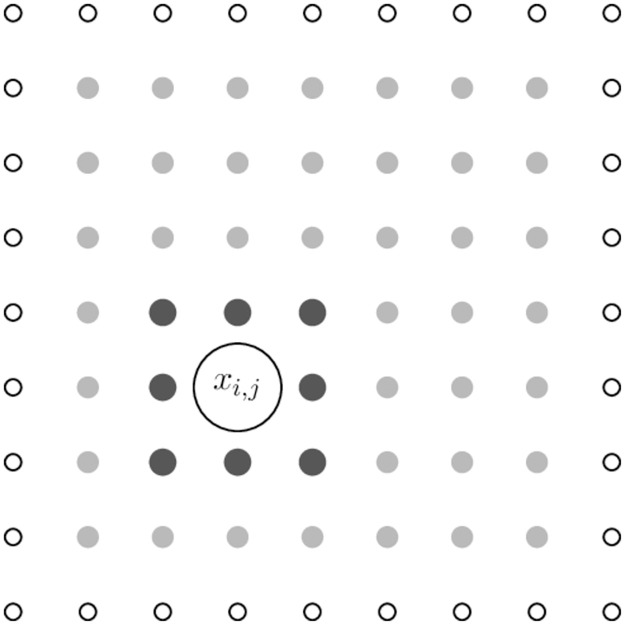
Random variates are created to form a grid of size *N* + 2 × *N* + 2 (white, hollow dots). At each of the *N* × *N* inner grey dots *ρ*_0_ is calculated by averaging the value of *α* across the nine neighbouring points on the lattice. At some *x*_*i*,*j*_ = (*x*_*i*_, *y*_*j*_) the neighbouring points are shown larger and in dark grey.

The above procedure is best visualised as in Fig 3.

**Fig 4:**
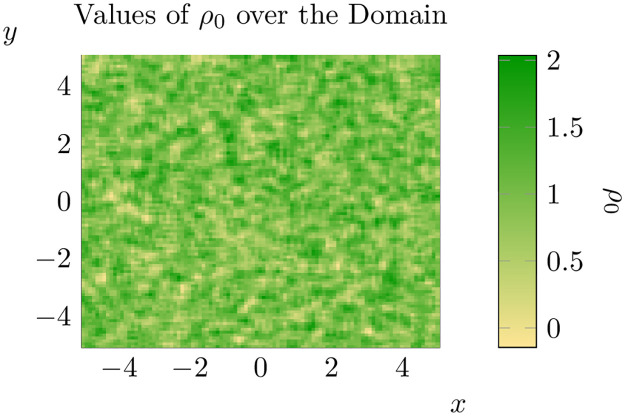
A typical resolution of the random field.

Following this procedure implies that the value of *ρ*_0_ at some point on the lattice is a random variable with mean 1 and standard deviation 1/3. Furthermore each variable is correlated with its neighbours, such that the lattice varies somewhat smoothly with *x* and *y* (see Fig 4 for an example of a typical resolution).

### 2.4 Simulation of an invasion with random *ρ*_0_

Before proceeding to solve the model on the ‘landscape’ we generated in the previous section, there are a number of practical issues that must be addressed. Firstly, we have allowed *ρ*_0_ to be negative, at least for isolated locations on the lattice. Since *ρ*_0_ is the linearised rate of growth for adult biomass, we will interpret this scenario as corresponding to the case where a location is not suitable for pine trees (e.g. is rocky or contains a small body of water). While we might be concerned about *A*(*t*, *x*, *y*) becoming negative in such locations, as long as *C*(*t*, *x*, *y*) ≥ 0 everywhere we are guaranteed that when *A*(*t*, *x*, *y*) = 0 then $\frac{\partial A}{\partial t}\geq 0$. A second, separate issue might occur if *A*(*x*, 0)>1 (which is to say, if the initial conditions were such that the adult population exceeded the equilibrium carrying capacity), but for the initial conditions we consider this can be neglected. Nonetheless, for more general initial conditions it might be necessary to truncate the distribution of *ρ*_0_ so that is strictly positive (as is done in the following section).

Secondly, given the variation in *ρ*_0_ we may be concerned about the stability of a typical finite differences scheme. Fortunately, it is relatively straightforward to show that a forward Euler scheme (with a centred difference approximation of the spatial derivatives) is stable using Von Neumann analysis. Since spatial variation only occurs in the first equation (e.g. Eq 10) and *A* appears only in Eq 11 as part of a function bounded between [−1, 1], one can write Eq 11 as

$$\begin{array}{c} \frac{\partial C}{\partial t}=D(\frac{\partial^{2}C}{\partial x^{2}}+\frac{\partial^{2}C}{\partial y^{2}})-C+f\left(t,C,x\right), \end{array}$$ (13)

where *f* is a strictly bounded function. Given this reformulation, it can be shown that the appropriate discretisation of Eq 13 is then stable. Finally, once the stability of the numerical scheme for Eq 13 is established the stability of the scheme for Eq 10 is a direct consequence.

**Fig 5:**
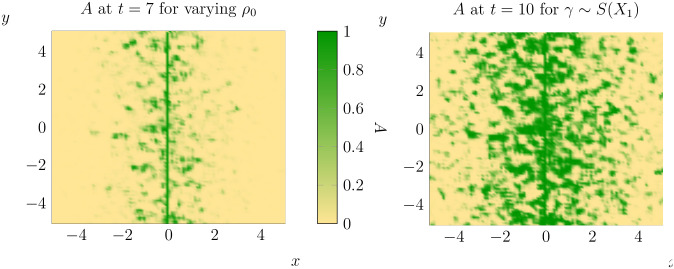
The state of *A* at *t* = 7 (left) and *t* = 10 (right) for the values of *ρ*_0_ given in Fig 4.

With these concerns out the way, we proceed to simulate an invasion with initial conditions Eq 8 over the ‘landscape’ shown in Fig 4. The state of this invasion at *t* = 7 and *t* = 10 is shown below (Fig 5). One can compare the above solution of the equation with randomly varying *ρ*_0_ to the case where *ρ*_0_ is set at the mean value across the entire domain (see Fig 2). Unsurprisingly there are significant differences between the outcomes of the proceeding two simulations. While there are a number of different dimensions along which one could compare these two simulations, we will focus on understanding how spatial heterogeneity changes the spread rate of pine trees. Of course, this requires us to define a notion of ‘spread rate’ that can be measured in our computational simulations.

### 2.5 Spread rates

**Fig 6:**
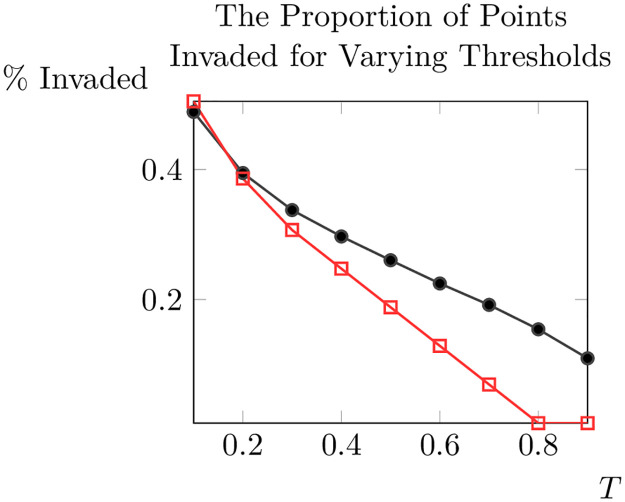
The proportion of points (*x*_*i*_, *y*_*j*_) on the lattice with *A*(*x*_*i*_, *y*_*j*_, 10) > *T* for varying *T*, with results for a constant value of *ρ*_0_ (red squares) and results for randomly generated values of *ρ*_0_ (black circles).

We will consider a point on the lattice (*x*_*i*_, *y*_*j*_) ‘invaded’ at *t* = 10 if *A*(*x*_*i*_, *y*_*j*_) > *T*, where *T* is some threshold between zero and one. We adopt this per-cell approach as it allows us to compute statistics on the evolution of the invasion without needing to identify a specific invasive front, which is obviously complicated by spatial heterogeneity. One can now consider the percent of cells on the lattice that are invaded for various thresholds for a constant value of *ρ*_0_ and for a realisation of our random field (see Fig 6).

As we can see, at least for this single realisation of the random distribution of *ρ*_0_, for the thresholds considered the proportion of invaded cells in the random case is equal to or higher than the proportion in the uniform case (except at *T* = 0.1 where the proportion invaded is slightly lower). Given this evidence that our spread-speed results are unlikely to be significantly different for different thresholds greater than 0.1, for the rest of this section we will consider a fixed threshold *T* = 0.5 (note that when considering the time when invasions are initiated, as we do below, results may be different for different thresholds). In [39] the authors also consider the proportion of cells or points invaded as a measure of invasion success, but since they do not report the criteria they use to determine whether a cell is invaded we cannot compare our approach to that of [39].

**Fig 7:**
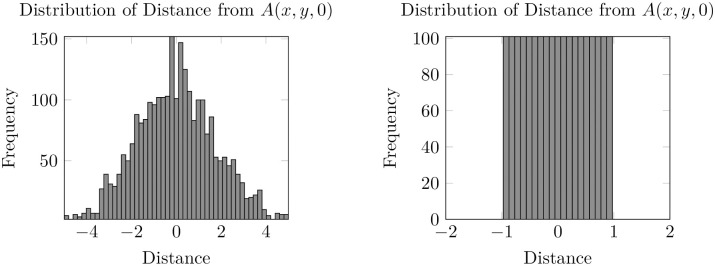
Histogram of the distribution of final signed distances for varying *ρ*_0_ (left). Histogram of the distribution of final signed distances for constant *ρ*_0_ (right).

At some time t_*_ one can consider the distribution of the signed distance of invaded cells (as defined in the sense above) to the initial condition ±‖(*x*_*i*_, *y*_*j*_) − proj_*A*(*x*, *y*,0)_(*x*_*i*_, *y*_*j*_)‖_2_ = *x*_*i*_. For t_*_ = 10 one can obtain the following histograms for varying *ρ*_0_ and constant *ρ*_0_ = 1 (see Fig 7).

**Fig 8:**
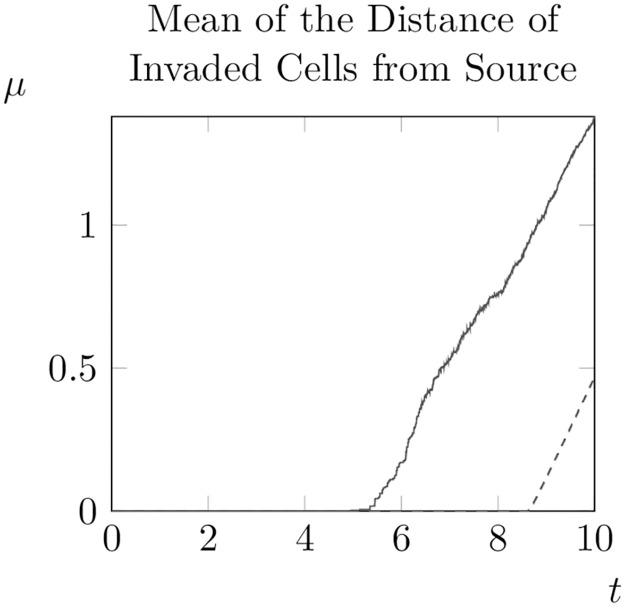
Evolution of the mean of the distance from the origin over an invasion for varying *ρ*_0_ (solid line) and constant *ρ*_0_ = 1 (dashed line). For the constant case Δ_*x*_ was set to 10^−2^ to eliminate discretisation artefacts (results were otherwise similar for Δ_*x*_ = 10^−1^).

As well as taking snapshots of the distribution for fixed *t*, one can also consider the evolution of the moments of the distribution of unsigned distances as *t* increases (we move from the signed distance to the unsigned distance so that the value of the first moment is not identically zero for all *t*). In particular, we consider the evolution of the first two moments of this distribution for varying and constant *ρ*_0_ (see Fig 8). Note that for larger values of Δ_*x*_ the evolution of the mean distance appears jagged due to the grid discretisation, but reducing the spatial step size eliminates this effect.

As can be seen, when *ρ*_0_ varies randomly the total proportion of the domain invaded at *t* = 10 is higher than for a constant value of *ρ*_0_. Furthermore, the average distance of invaded cells from the initial condition and the variance of the distribution of distances was much higher when *ρ*_0_ varied randomly. Thus, in this particular realisation of *ρ*_0_, we can see that allowing *ρ*_0_ to vary increases the spread of adult pine trees (at least at *t* = 10).

### 2.6 Inferring spatial spread rates

One natural question to ask is how the time evolution of the moments of these distributions are related to the asymptotic spread rates that have been computed for previous models. Consider first the case where *ρ*_0_ is constant across the domain. In this case *A* and *C* are constant with respect to *y*, so we expect that the solution to our model will depend only on *x* and *t*. At least for constant values of *ρ*_0_ we expect that 2D dynamics will closely resemble those of the 1D model and thus based on Fig 1 and previous work (see also S1 Fig for a plot over a longer time period) we expect that after some critical time t_*_ (with t_*_ ≈ 7 < 10) the solution for *A* will approach a travelling wave *f*(|*ξ*|), *ξ* = *x* − *st*, with spread rates approaching a constant rate before *t* < 10 (see [36]). Given this, we expect that for *t* > t_*_ we can write the set of ‘invaded’ points (those points such that *A*(*t*, *x*, *y*) > *T* for some *T* ∈ [0, 1]) as

$$\begin{array}{c} I=\left[-a-st,a+st\right]\times \left[-5,5\right],a\in R^{+}, \end{array}$$ (14)

for some a,s∈R. A direct implication is that at time *t* every point with distance to the initial condition less than *a* + *st* − *L*/*n* is invaded. This suggests that the distribution of distances to the initial condition at time *t* should be approximately uniform (except for an isolated peak at 0, as the initial condition has positive measure). The probability of a randomly selected point landing in the initial condition must thus be (2*L*/*n*)/(2*a* + 2*st*) = *L*/(*n*(*a* + *st*)) and thus decreases monotonically as the invasion proceeds. Therefore for large *n* and *t* we expect the distribution to be approximately uniform and have mean

$$\begin{array}{c} \mu \left(t\right)=\frac{1}{2}\left(a+st\right). \end{array}$$ (15)

So the average distance of invaded points from the origin moves outwards at a constant speed *s*/2 which is half the speed of the hypothetical travelling wave solution. Given this, we fit a piecewise linear function to the curves shown in Fig 8

$$\begin{array}{c} \hat{\mu }\left(t\right)=\text{max}\left\{0,\alpha +\beta t\right\}. \end{array}$$ (16)

**Fig 9:**
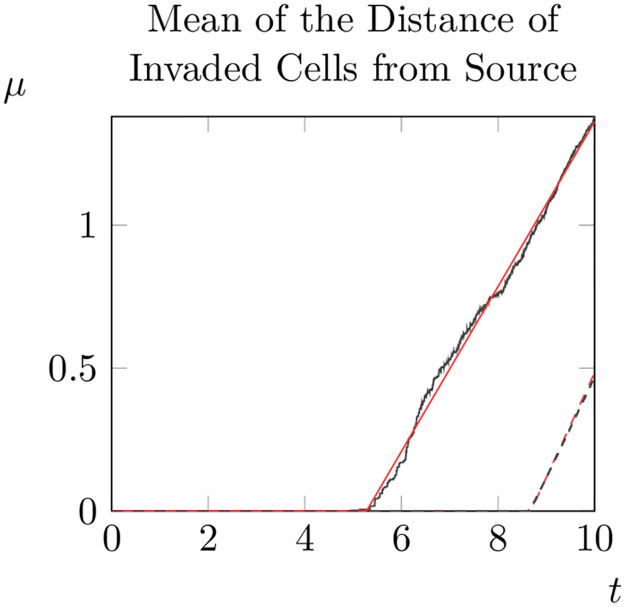
The mean of distance of ‘invaded’ points from the initial condition for varying *ρ*_0_ (black solid lines) and a linear fit of the mean distance (red solid lines). The corresponding data for constant *ρ*_0_ is shown with dashed lines.

Plotting the results (see Fig 9), we can see that our fitted function closely matches the actual increase in *μ* over time. We can also apply this method to obtain an estimate for the spread rate for randomly generated *ρ*_0_.

Since this linear function is a good fit for the data, this suggests that for homogeneous *ρ*_0_ and parameter values as in [36] the spread speed of invasions is *s* ≈ 0.72. Furthermore, for this particular set of random values of *ρ*_0_ we have *s* = 0.62. This suggests that in this case, the spread rate for this particular set of randomly generated values of *ρ*_0_ is slightly less than in the case of constant of *ρ*_0_. However, since spread occurs much earlier in the case where *ρ*_0_ varies the mean distance is much higher when *t* = 10 than the case when *ρ*_0_ is constant.

Returning to the linear case, to confirm that this spread speed was not an artefact of the exact spacing of the spatial discretisation we used, we also simulated an invasion with ten different values of Δ_*x*_ between 1/9 and 1/11. Then, taking the evolution of the mean distance of invaded points to the initial condition from each ‘invasion’ we fitted our piecewise linear function to the resulting ≈10^5^ points, obtaining an average spread rate of *s* ≈ 0.72. This provides evidence that our estimated spread rate was not an artefact of the particular spatial discretisation we used.

**Fig 10:**
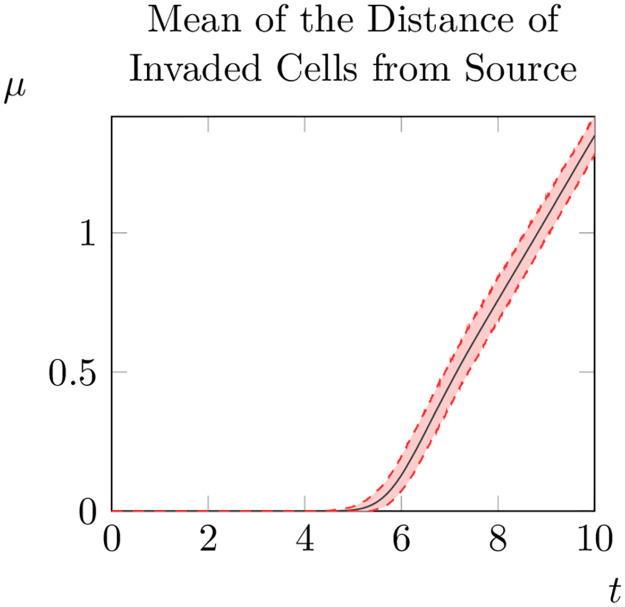
Averaged evolution of the mean distance of invaded cells to the initial condition across one thousand iterations.

Of course, results about the relative spread speed for constant and varying *ρ*_0_ could be an outlier and not reflect the average outcome over a larger number of realisations of the random variable *ρ*_0_(*x*_*i*_, *y*_*j*_). To consider this possibility we repeated one thousand iterations of our model from *t* = 0 to *t* = 10, computing at each timestep the average distance of invaded points to the initial condition. Then, for each point in time we averaged across all iterations to obtain an average-average distance to the initial condition curve (see Fig 10). As a measure of the variability across iterations, for each timestep we also plot the 95% interval of average distances (e.g. the interval between the average distance at the 97.5-th percentile and the 2.5-th percentile). Interestingly, we observe that these curves are largely parallel to the average-average distance curve, suggesting that variation is largely driven by differences in ‘take-off’ time (see Fig 10).

We then construct a piecewise linear fit to obtain an estimate for the average spread rate as above. In this case *s* ≈ 0.60, suggesting that the spread rate for randomly generated *ρ*_0_ will be slower than for constant *ρ*_0_. However, because ‘take off’ appears to occur earlier when *ρ*_0_ is generated randomly, the mean distance of invaded points at *t* = 10 is larger for randomly generated *ρ*_0_ than for constant *ρ*_0_. Given that introducing random spatial variation in at least one parameter in the system can have a significant impact on the dynamics on the system, we might also naturally wonder whether spatial variation in any of the other parameters produces equally important changes in dynamics.

In particular, as well as *ρ*_0_ (which governs growth of populations after establishment) we will also consider changes in seed production and in the carrying capacity of environments. Given that in the previous section we allow the growth rate of trees to vary but keep carrying capacity fixed, it is natural to investigate how holding the growth rates constant while allowing carrying capacity to vary may impact spread. In ecological terms, varying *ρ*_0_ could represent a situation where resource quality varies across a landscape, while varying carrying capacity varies resource availability. The fecundity of adult trees is a natural factor to vary, given that variation in seed production is well known to vary significantly between trees [40].

Variation in the population growth (e.g. *ρ*_0_) is easy to introduce (see the previous section). To vary the carrying capacity we must first note that, in our model, the carrying capacity K_*_ of a particular location is the equilibrium amount of adult biomass in that location, or

$$\begin{array}{c} K_{*}=1+O\left(\epsilon \right). \end{array}$$ (17)

To allow for spatial variation, we shall introduce the parameter *κ*, so that the equation for *A* becomes

$$\begin{array}{c} \frac{\partial A}{\partial t}=\epsilon C+\rho_{0}A-\kappa A^{2}, \end{array}$$ (18)

Where *ϵ* ∼ 10^−2^ and *ρ*_0_, *κ*, *C* ∼ 1. The carrying capacity now becomes $K_{*}=\rho_{0}/\kappa +O\left(\epsilon \right)$ so to impose spatial variation in K_*_ without affecting the establishment rate of adult trees, we shall allow *κ* to vary spatially. Finally, to vary seed production by location we will introduce the parameter *γ* and allow it to vary spatially so that Eq 11 becomes

$$\begin{array}{c} \frac{\partial C}{\partial t}=D(\frac{\partial^{2}C}{\partial x^{2}}+\frac{\partial^{2}C}{\partial y^{2}})-C+\gamma \frac{A^{2}}{\beta^{2}+A^{2}}. \end{array}$$ (19)

In [35] it has been demonstrated that different distributional assumptions on parameters can significantly impact outcomes (at least in some models), we will compare the impact of allowing each of the parameters to vary under three different distributional assumptions. Before proceeding further we will establish a little notation. Firstly, let us denote the spatial averaging operation discussed in subsection 2.3 as *S*, so that the spatially averaged random variable discussed in the previous section is *S*(*Z*) (where *Z* is a normally distributed random variable with mean one and standard deviation one). Then we will consider the following nine possible combinations of variables and distributions given in Table 2.

**Table 2:** Combinations of distributions and parameters considered (note that truncnorm(*a*, *b*, *c*, *d*) refers to a truncated normal distribution with bounds *a* and *b* and with *μ* = *c*, *σ*^2^ = *d*).

	*X*_1_ = truncnorm(0.1, 1.9, 1, 0.3)	*X*_2_ = uniform(0.1, 1.9)	*X*_3_ = exp(0.9) + 0.1
*ρ*_0_	*ρ*_0_ ∼ *S*(*X*_1_)	*ρ*_0_ ∼ *S*(*X*_2_)	*ρ*_0_ ∼ *S*(*X*_3_)
*κ*	*κ* ∼ *S*(*X*_1_)	*κ* ∼ *S*(*X*_2_)	*κ* ∼ *S*(*X*_3_)
*γ*	*γ* ∼ *S*(*X*_1_)	*γ* ∼ *S*(*X*_2_)	*γ* ∼ *S*(*X*_3_)

Note that all parameters have been chosen so that these distributions are bounded below by 0.1 (since *κ* or *γ* are bounded below at zero by biological constraints) and are such that *E*(*S*(*X*_*i*_)) = 1 for all *i*. However, we expect that the uniform and exponential distributions will produce more extreme values than the truncated normal distribution, with the exponential distribution having a particularly high chance of producing large positive outliers. In ecological terms this corresponds to increasing spatial heterogeneity, with greater variation in the local site attributes.

## 3 Results

**Fig 11:**
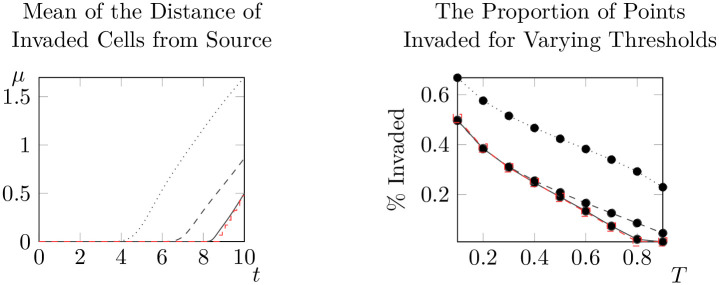
Mean distances from invaded cells to the initial condition over time (left) and proportion of points invaded at *t* = 10 for differing thresholds (right) for *ρ*_0_ ∼ *S*(*X*_1_) (solid black line), *ρ*_0_ ∼ *S*(*X*_2_) (dashed black line), *ρ*_0_ ∼ *S*(*X*_3_) (dotted black line) and *ρ*_0_ = 1 (dashed red line).

**Fig 12:**
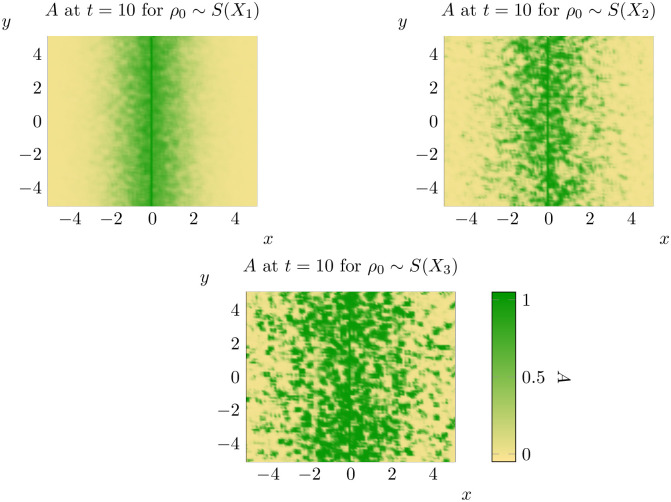
Representative states for *ρ*_0_ ∼ *S*(*X*_1_) (top left), *ρ*_0_ ∼ *S*(*X*_2_) (top right) and *ρ*_0_ ∼ *S*(*X*_3_) (bottom) at *t* = 10. See bottom left for the scale for all three states.

**Fig 13:**
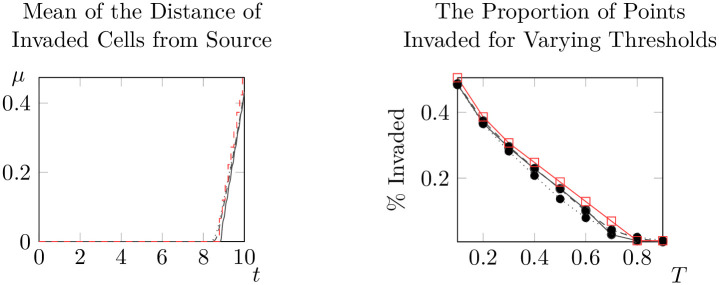
Mean distances from invaded cells to the initial condition over time (left) and proportion of points invaded at *t* = 10 for differing thresholds (right) for *κ* ∼ *S*(*X*_1_) (solid black line), *κ* ∼ *S*(*X*_2_) (dashed black line), *κ* ∼ *S*(*X*_3_) (dotted black line) and *κ* = 1 (dashed red line).

**Fig 14:**
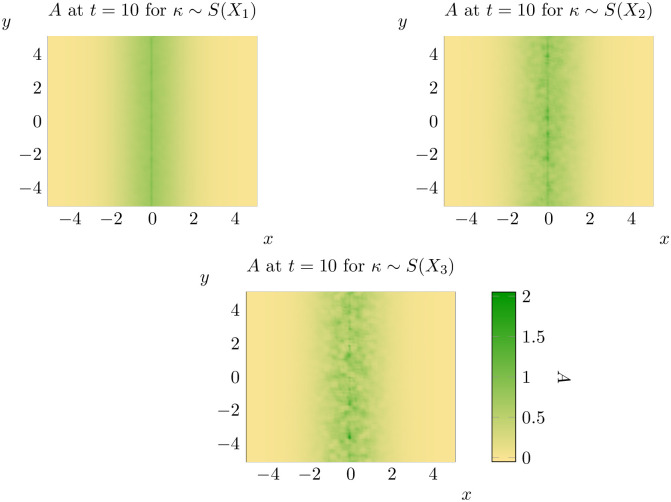
Representative states for *κ* ∼ *S*(*X*_1_) (top left), *κ* ∼ *S*(*X*_2_) (top right) and *κ* ∼ *S*(*X*_3_) (bottom) at *t* = 10. See bottom left for the scale for all three states (note that this is different to that of the previous two figures).

**Fig 15:**
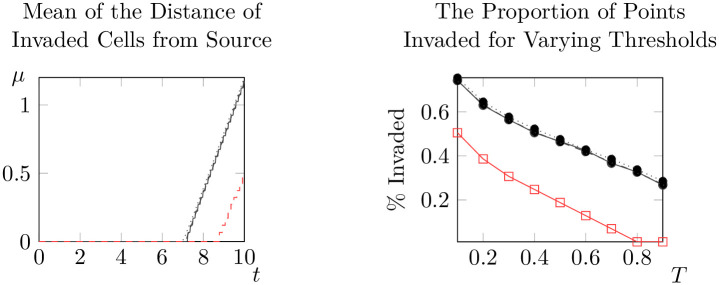
Mean distances from invaded cells to the initial condition over time (left) and proportion of points invaded at *t* = 10 for differing thresholds (right) for *γ* ∼ *S*(*X*_1_) (solid black line), *γ* ∼ *S*(*X*_2_) (dashed black line), *γ* ∼ *S*(*X*_3_) (dotted black line) and *γ* = 1 (dashed red line).

**Fig 16:**
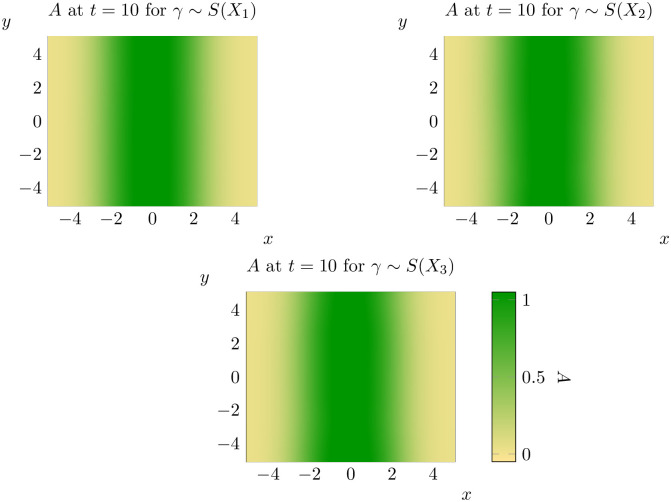
Representative states for *γ* ∼ *S*(*X*_1_) (top left), *γ* ∼ *S*(*X*_2_) (top right) and *γ* ∼ *S*(*X*_3_) (bottom) at *t* = 10. See bottom left for the scale for all three states.

For each of these nine combinations, we will compute the spread rate discussed in the previous section and the average percentage of points invaded for different thresholds at *t* = 10. Beginning first with *ρ*_0_ ∼ *S*(*X*_1_) we consider each of these nine combinations in turn (see below for Figs 11 to 16).

## 4 Discussion

A few trends are clear from the results of our experiments. Firstly, it is important to note that the impact of spatial variation in a parameter is significantly mediated by the combination of parameter and distribution chosen. Spatial variation in *ρ*_0_ generally causes spatial spread to begin earlier, but spread rates are sometimes lower than with *ρ*_0_ constant. Variation in *γ* leads to faster onset of spatial spread and faster spatial spread rates, but changing distributional assumptions do not appear to significantly alter outcomes. Notably, the qualitative state of the solution as *t* = 10 is not significantly different from the case of constant *γ* under all three distributional assumptions. Changing distributional assumptions for *κ* had a significant impact on the percentage of points invaded at *t* = 10, but not on the spatial spread rate or the value at which spatial spread began. The spatial spread rates and largest values of *t* at which $\hat{\mu }\left(t\right)=0$ (that is, the time at which spread begins) are recorded in Tables 3 and 4 respectively. Values in brackets give the corresponding estimates for the 95% interval of each of these parameters. However, faster spread rates are not necessarily associated with faster take-off times, so these values cannot be combined to estimate bounding curves like those in Fig 10. For bounding curves and enlarged versions of these plots, see supplementary information (S2 to S10 Figs).

**Table 3:** Spatial spread rates for each of the combinations of parameters and distributions considered (rounded to two decimal places).

	*X*_1_ = truncnorm(0.1, 1.9, 1, 0.3)	*X*_2_ = uniform(0.1, 1.9)	*X*_3_ = exp(0.9) + 1
*ρ*_0_	0.62 [0.58, 0.66]	0.54 [0.49, 0.61]	0.62 [0.57, 0.68]
*κ*	0.72 [0.71, 0.73]	0.63 [0.60, 0.67]	0.58 [0.54, 0.64]
*γ*	0.82 [0.82, 0.82]	0.81 [0.81, 0.81]	0.81 [0.79, 0.81]

**Table 4:** Times of the onset of spatial spread for each of the combinations of parameters and distributions considered (rounded to two decimal places).

	*X*_1_ = truncnorm(0.1, 1.9, 1, 0.3)	*X*_2_ = uniform(0.1, 1.9)	*X*_3_ = exp(0.9) + 1
*ρ*_0_	8.41 [7.29, 9.70]	6.83 [5.34, 8.70]	4.31 [3.41, 5.43]
*κ*	8.82 [8.57, 9.07]	8.86 [7.70, 9.74]	8.58 [7.18, > 10]
*γ*	7.13 [7.12, 7.15]	7.12 [7.05, 7.20]	7.02 [6.84, 7.23]

Despite the significant impacts of spatial variation, all combinations of parameters and distributions continued to display evidence of solutions that do not significantly evolve until a critical time t_*_, before rapidly evolving towards solutions which advance at a constant rate (at least with respect to the mean distance of invaded locations to the source population). The possible exception to this rule is *ρ*_0_ ∼ *X*_3_, where it appears that the rate at which $\hat{\mu }\left(t\right)$ increases is decreasing for *t* ∼ 10. However, even in this case our results suggest that spread rates are almost constant for *t* ∼ 1. This suggests that the model of [36] is at least qualitatively robust to random variation in parameters.

**Fig 17:**
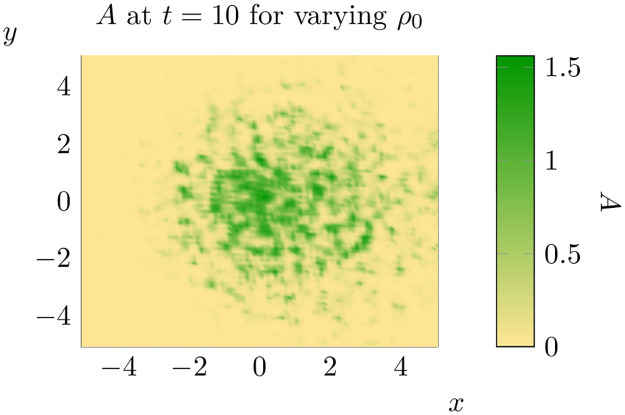
The distribution of *A* at *t* = 10 for a model with an O(1) advective term (*ν* ≈ (1, 0)) and *ρ*_0_ ∼ *S*(*X*_1_). Note that the initial condition in this case is a circular ‘blob’ of width 0.4 rather than a thin line as in previous plots.

Furthermore, at least in some cases, the solutions obtained appear to generate clusters of population growth like those observed in invaded landscapes (compare Fig 12 to Fig 1 in [14] for example). Spatial variation in one or more parameters can be combined with other extensions to this model to produce spread patterns that are even closer to those observed in invaded landscapes. If one considers the advective model advanced in [36] but with spatial variation in *ρ*_0_ this leads to the following equation

$$\begin{array}{c} \frac{\partial A}{\partial t}=\epsilon C+\rho_{0}\left(x,y\right)A\left(1-A\right), \end{array}$$ (20)

$$\begin{array}{c} \frac{\partial C}{\partial t}=D\nabla^{2}C+\nu \cdot \nabla C-C+\frac{A^{2}}{\beta^{2}+A^{2}}, \end{array}$$ (21)

and one can produce patterns which are semi-random and exhibit a preferred direction of spread (see Fig 17).

## 5 Conclusion

Many models of invasive species spread are deterministic, while the actual invasion process is stochastic. For example, seed production appears to be approximately negative binomial distributed among invasive trees [40], so in practice *γ* may vary stochastically between different locations in the landscape. While there is some evidence from similar environments overseas that heterogeneity in site suitability may be overwhelmed by the dispersal process [20], this does not guarantee that random variation in *γ* (e.g. seed production) or other parameters do not impact spread.

To explore this possibility, we considered extensions to a recent model for exotic conifer invasion [36] which allowed a parameter to vary randomly. We showed that, under a variety of parameter choices and distributional assumptions, solutions continued to exhibit a constant spread rate (measured by the evolution of the average distance between a unit of biomass and the initial condition). Qualitatively and quantitatively, there were significant differences between solutions depending upon the assumptions made, with differences in both the asymptotic spread rate and the time at which spatial spread began. Some choices of parameters led to significant increases in the spread rate but produced solutions that appeared qualitatively similar to solutions arising from parameters which are constant everywhere. Other solutions produced similar spread rates but had a very different qualitative appearance. However, the robustness of the two-regime structure of the solution is promising, as it suggests that this model may be resilient to significant local variation in parameter values.

**Fig 18:**
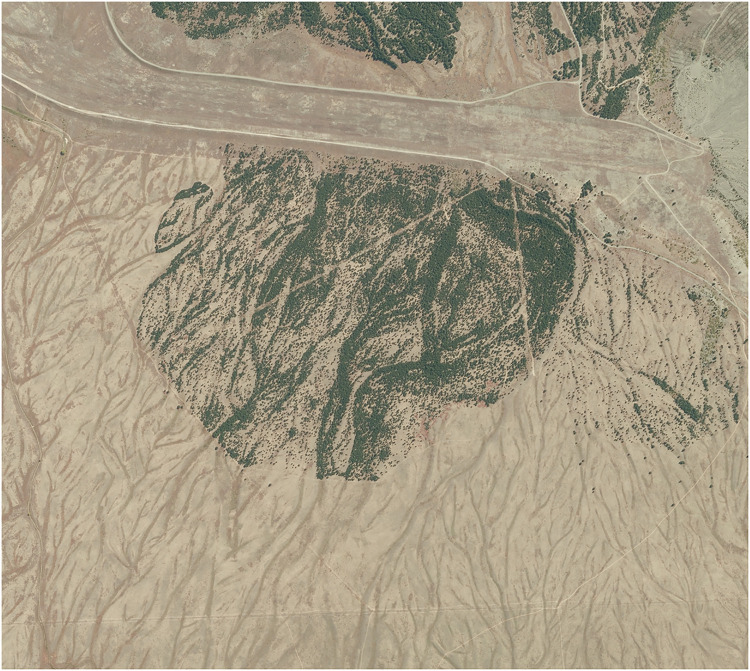
An aerial photo of an invasive population (note that the origin of this population appears to have been in the upper right-hand corner of this figure) from near Lake Pukaki, New Zealand. Further details and earlier images of this site can be found in [41]. Image sourced from the LINZ Data Service and licensed by The Canterbury Aerial Imagery (CAI) consortium for reuse under a CC BY 4.0 license.

Furthermore, in some cases we observe qualitative patterns that match those observed in New Zealand environments (e.g. highly clustered spread). Compare, for example, Figs 17 and 18.

This provides some evidence that a suitable version of our model may produce plausible qualitative pictures of invasions as well as estimates of spread rates or other quantities. This suggests that understanding the spatial patterns in parameters may improve our ability to accurately forecast spread. In some cases, it may be possible (e.g. by using homogenisation techniques) to ‘average out’ random variation and obtain accurate estimates (as discussed in [27] and other references above). In other cases it may be possible to obtain reasonably accurate models by neglecting spatial variation in some parameters if the variation does not have a significant impact on the solutions. Alternatively it may sometimes be necessary to explicitly include stochastic variation in parameters, although if many parameters are set to vary randomly an agent-based model may be a more appropriate choice. In any case, our modelling suggests that spatial heterogeneity may have an important impact on spread rates of pine trees and that future research should not ignore the possible impacts of random variation.

In order to fit this model (or other similar spatially explicit models) to data, it will be necessary to construct methods of generating landscapes that match the observed characteristics of areas vulnerable to invasion. This requires both detailed spatial data and ecological insight, as the relationship between observable variables and outcomes may not be straightforward (see [42] for further discussion). In some previous work, such as [30], model landscapes were constructed by replicating the characteristics of a particular location that previously experienced an invasion. Ultimately, however, it would be desirable to be able to construct landscapes which are like those observed without necessarily copying a particular landscape, so as to be able to make modelling predictions over a wider range of potential scenarios.

It also should be noted that, while we focused on spatial variation induced by random variation in the environment, other sources of spatial variation can also be ecologically significant. For example, future work on invasive conifers could explore the impact of different management strategies on spread (as in [15]) or environmental phenomena that also evolves over time (e.g. coinvasion by herbivores). These time-varying phenomena could potentially have significant impacts on the dynamics of solutions and insights from such models may have particularly valuable management implications, so future work in this area could be productive.

## Supporting information

S1 Fig Invasion dynamics from *t* = 0 to *t* = 20 on double-length domain. Note the clear emergence of travelling wave-type behaviour for larger *t* > t_*_ ≈ 7.
(TIF)

S2 Fig The mean and 95% intervals of the mean distance of invaded cells from the source across 1000 iterations, with *ρ*_0_ ∼ *S*(*X*_1_). (TIF)

S3 Fig The mean and 95% intervals of the mean distance of invaded cells from the source across 1000 iterations, with *ρ*_0_ ∼ *S*(*X*_2_). (TIF)

S4 Fig The mean and 95% intervals of the mean distance of invaded cells from the source across 1000 iterations, with *ρ*_0_ ∼ *S*(*X*_3_). (TIF)

S5 Fig The mean and 95% intervals of the mean distance of invaded cells from the source across 1000 iterations, with *κ* ∼ *S*(*X*_1_). (TIF)

S6 Fig The mean and 95% intervals of the mean distance of invaded cells from the source across 1000 iterations, with *κ* ∼ *S*(*X*_2_). (TIF)

S7 Fig The mean and 95% intervals of the mean distance of invaded cells from the source across 1000 iterations, with *κ* ∼ *S*(*X*_3_). (TIF)

S8 Fig The mean and 95% intervals of the mean distance of invaded cells from the source across 1000 iterations, with *γ* ∼ *S*(*X*_1_). (TIF)

S9 Fig The mean and 95% intervals of the mean distance of invaded cells from the source across 1000 iterations, with *γ* ∼ *S*(*X*_2_). (TIF)

S10 Fig The mean and 95% intervals of the mean distance of invaded cells from the source across 1000 iterations, with *γ* ∼ *S*(*X*_3_). (TIF)
