# Supplementary figures and images for: Partial differential equation models for invasive species spread in the presence of spatial heterogeneity
Source: PLoS One. 2024 Apr 2;19(4):e0300968. doi: 10.1371/journal.pone.0300968 (PMC10986974; doi:10.1371/journal.pone.0300968)

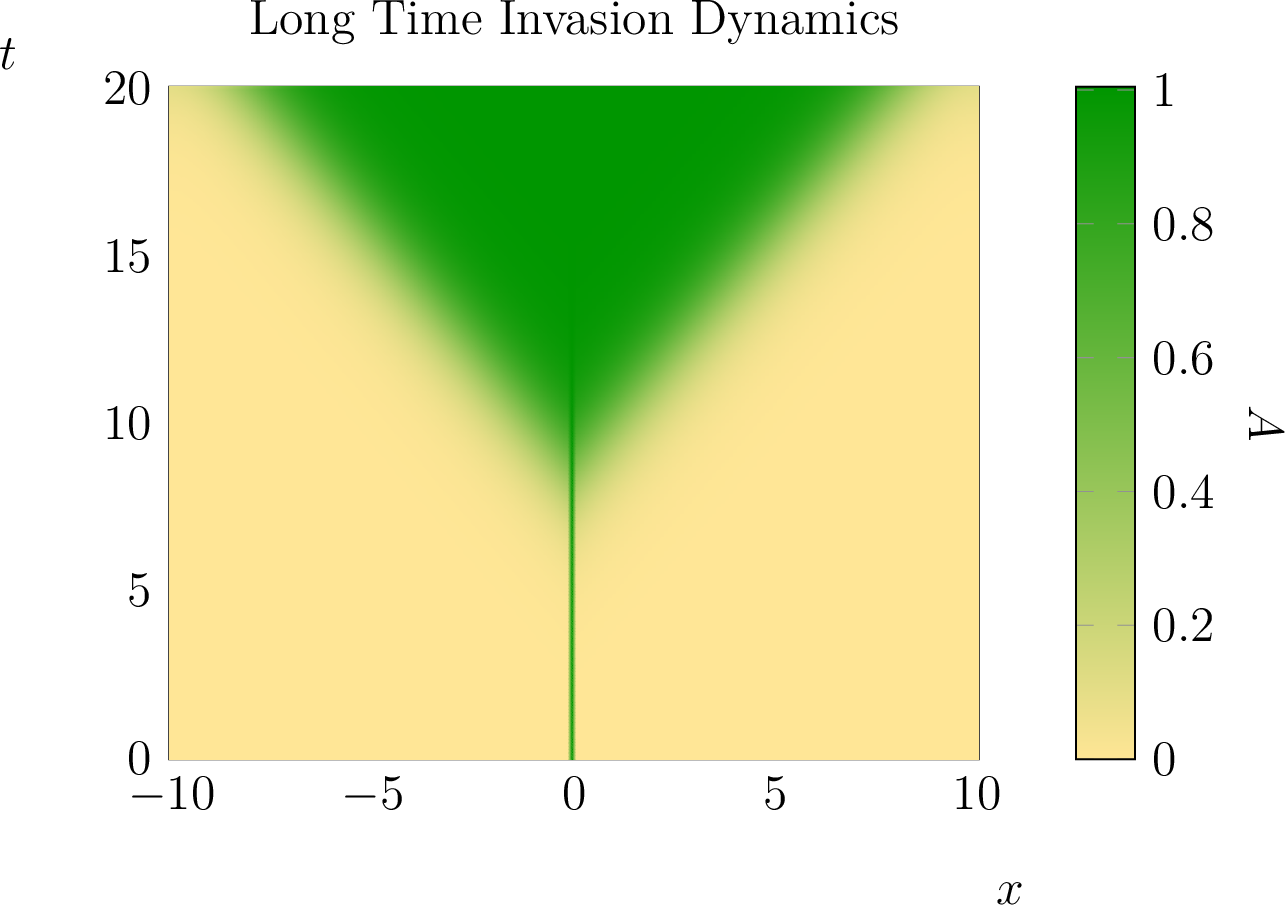

Supplement: S1 Fig — Note the clear emergence of travelling wave-type behaviour for larger t > t* ≈ 7. (TIF) [file pone.0300968.s001.tif]

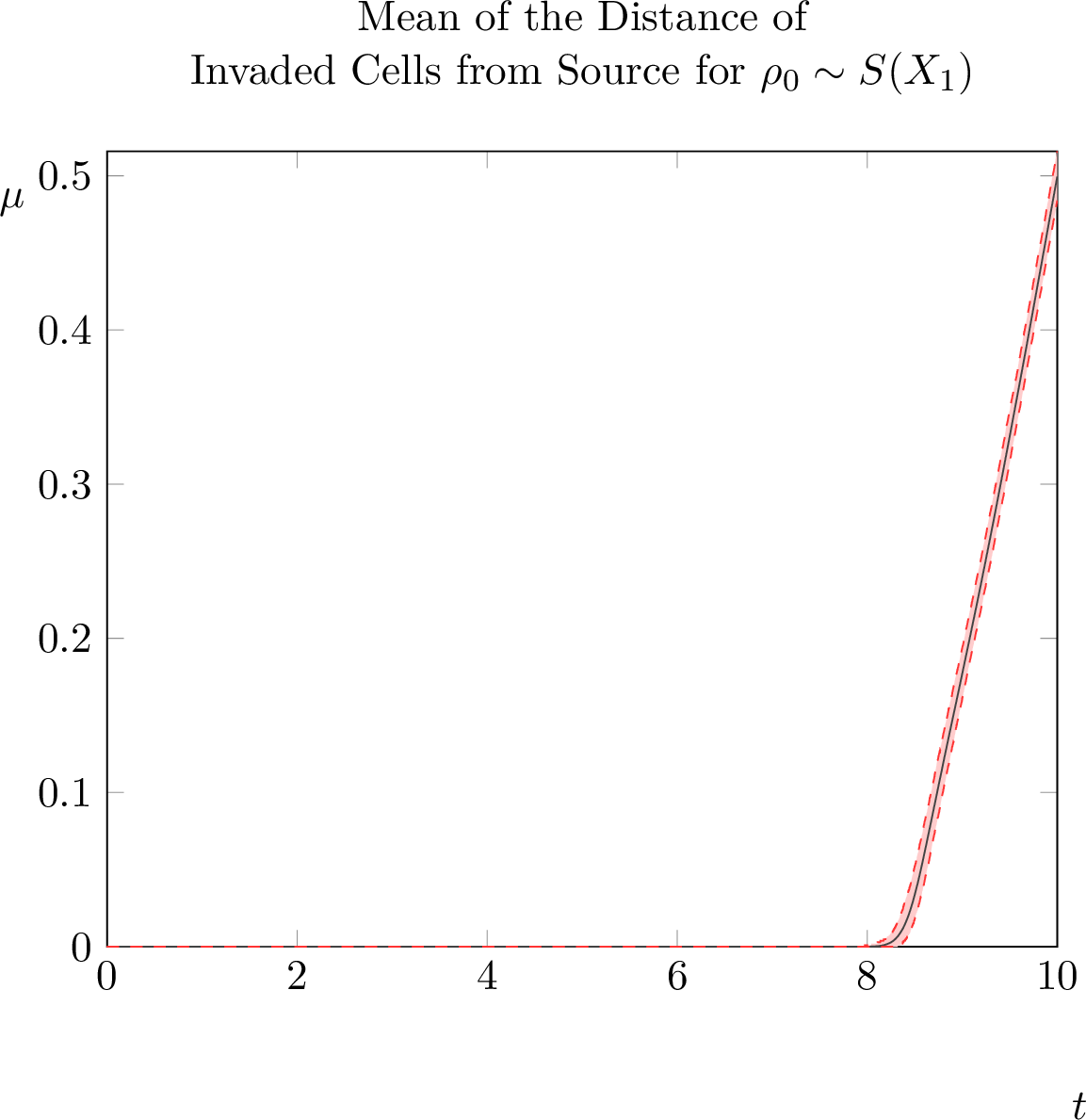

Supplement: S2 Fig — (TIF) [file pone.0300968.s002.tif]

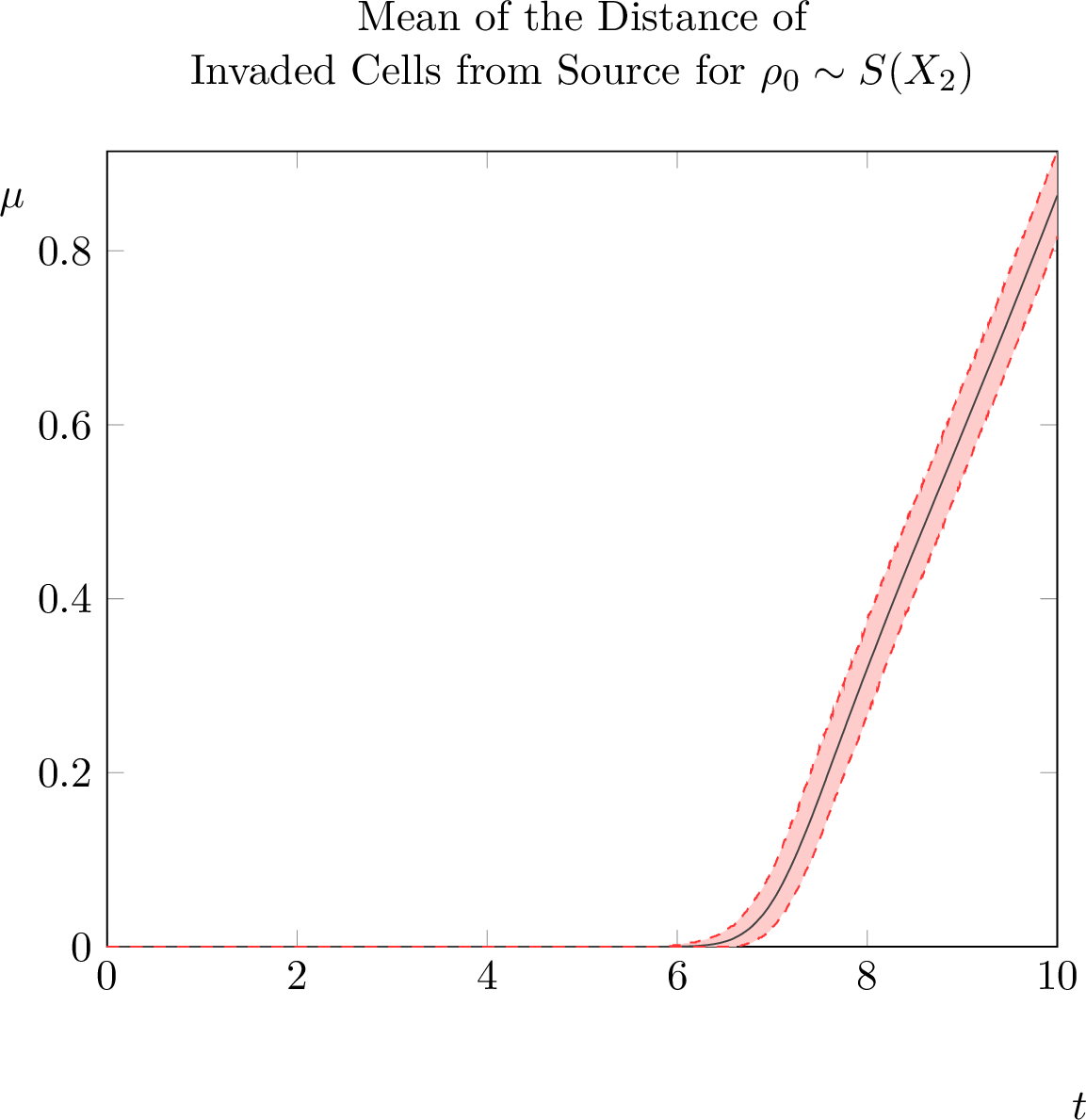

Supplement: S3 Fig — (TIF) [file pone.0300968.s003.tif]

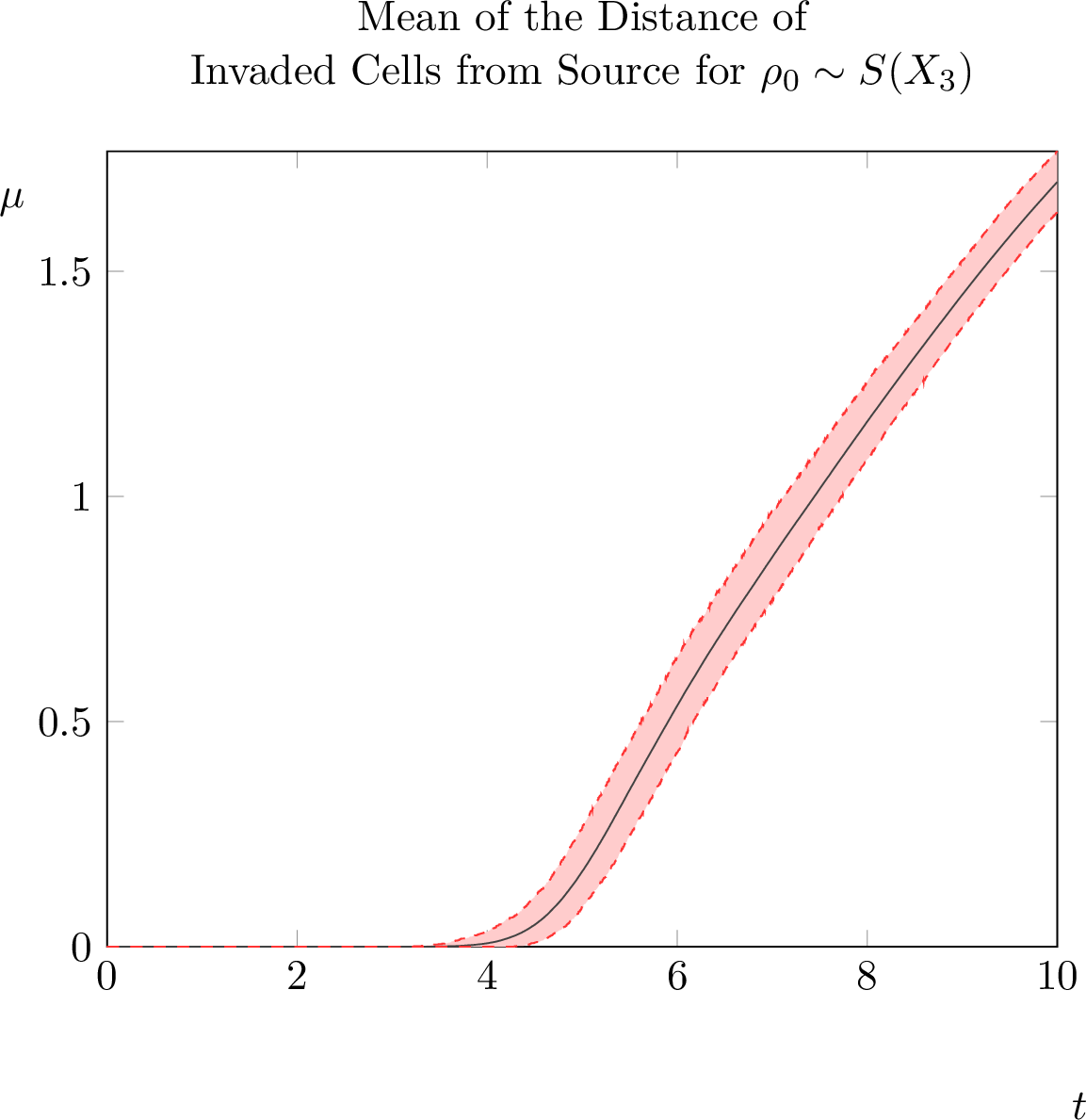

Supplement: S4 Fig — (TIF) [file pone.0300968.s004.tif]

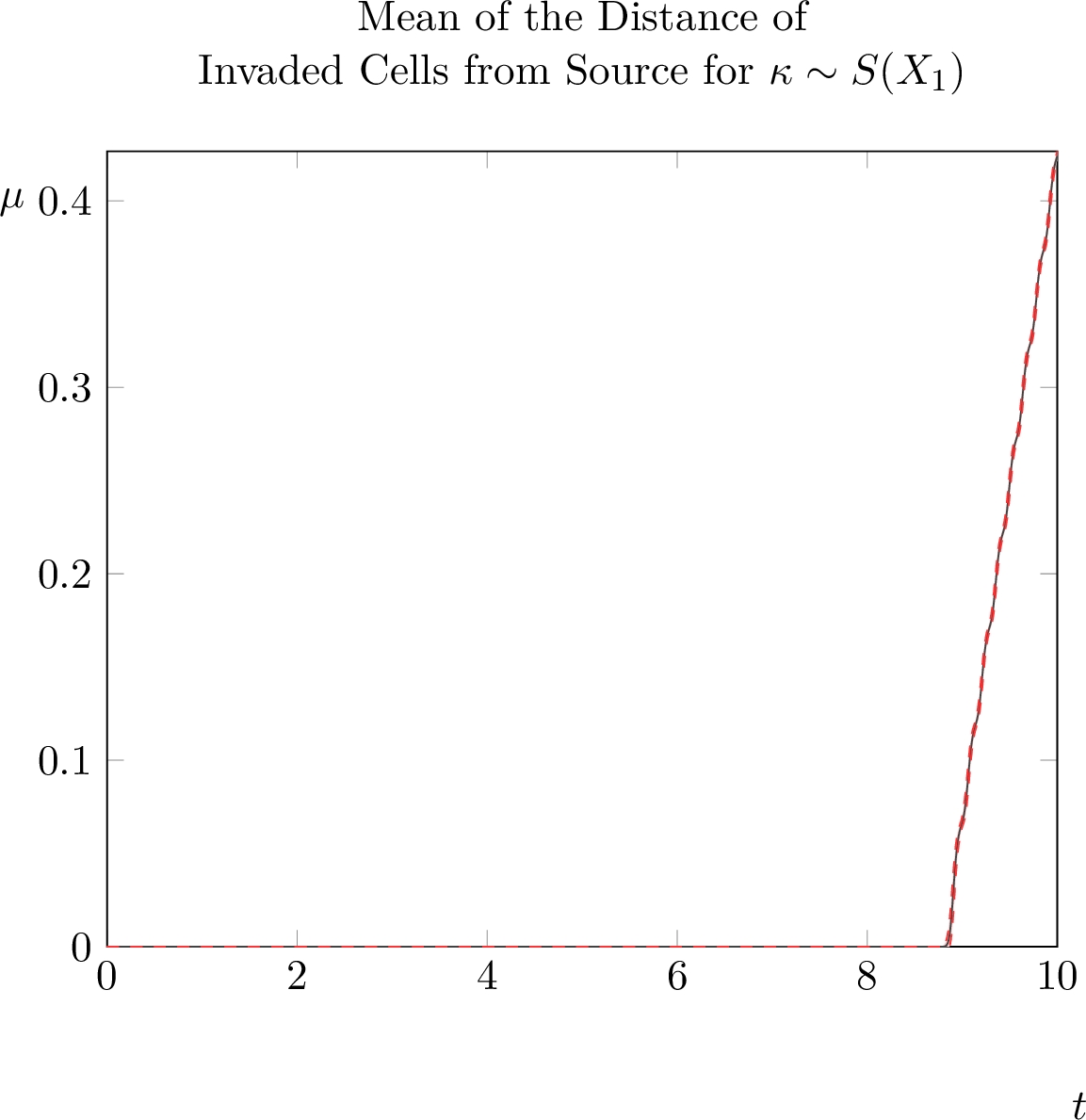

Supplement: S5 Fig — (TIF) [file pone.0300968.s005.tif]

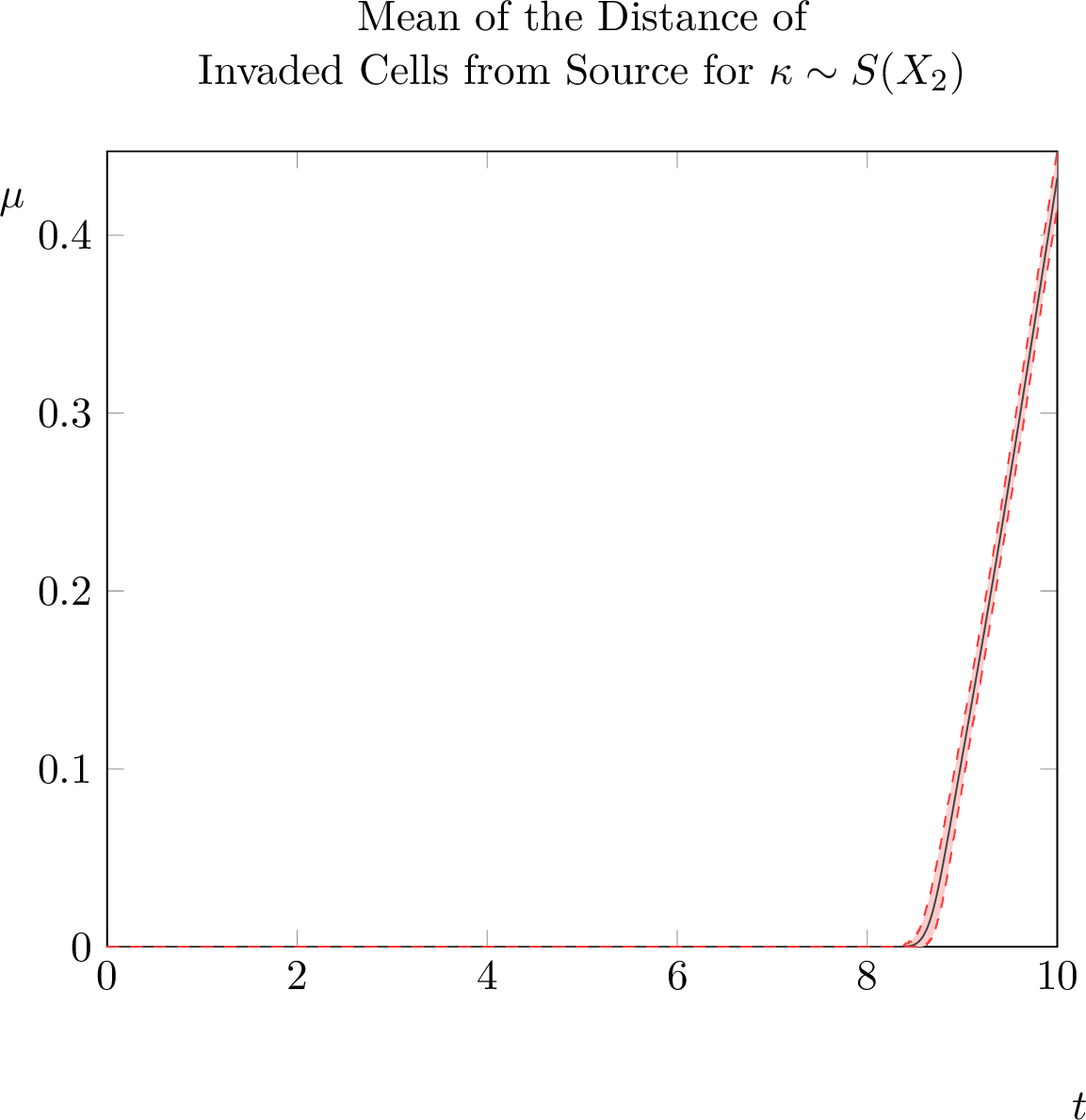

Supplement: S6 Fig — (TIF) [file pone.0300968.s006.tif]

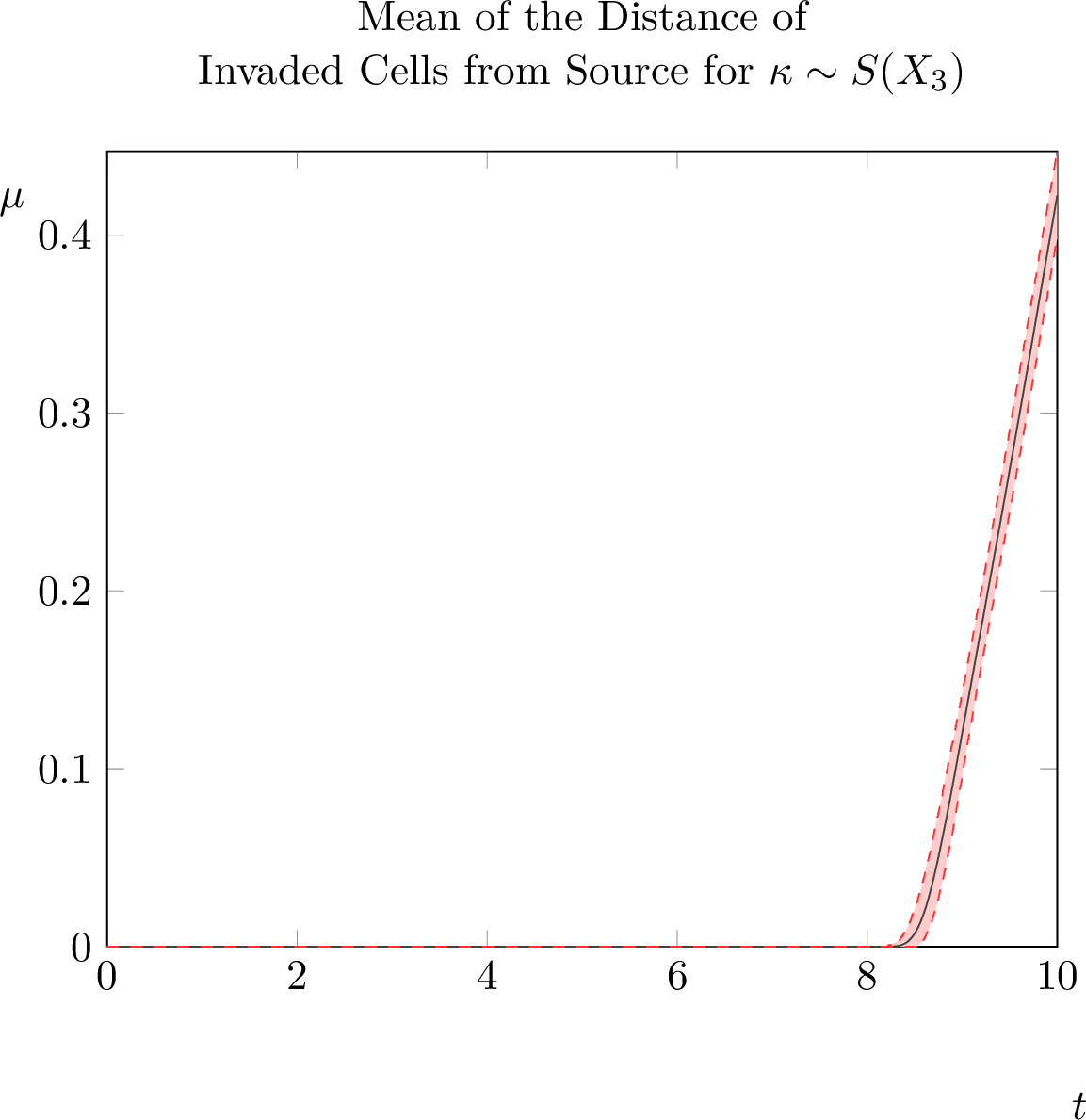

Supplement: S7 Fig — (TIF) [file pone.0300968.s007.tif]

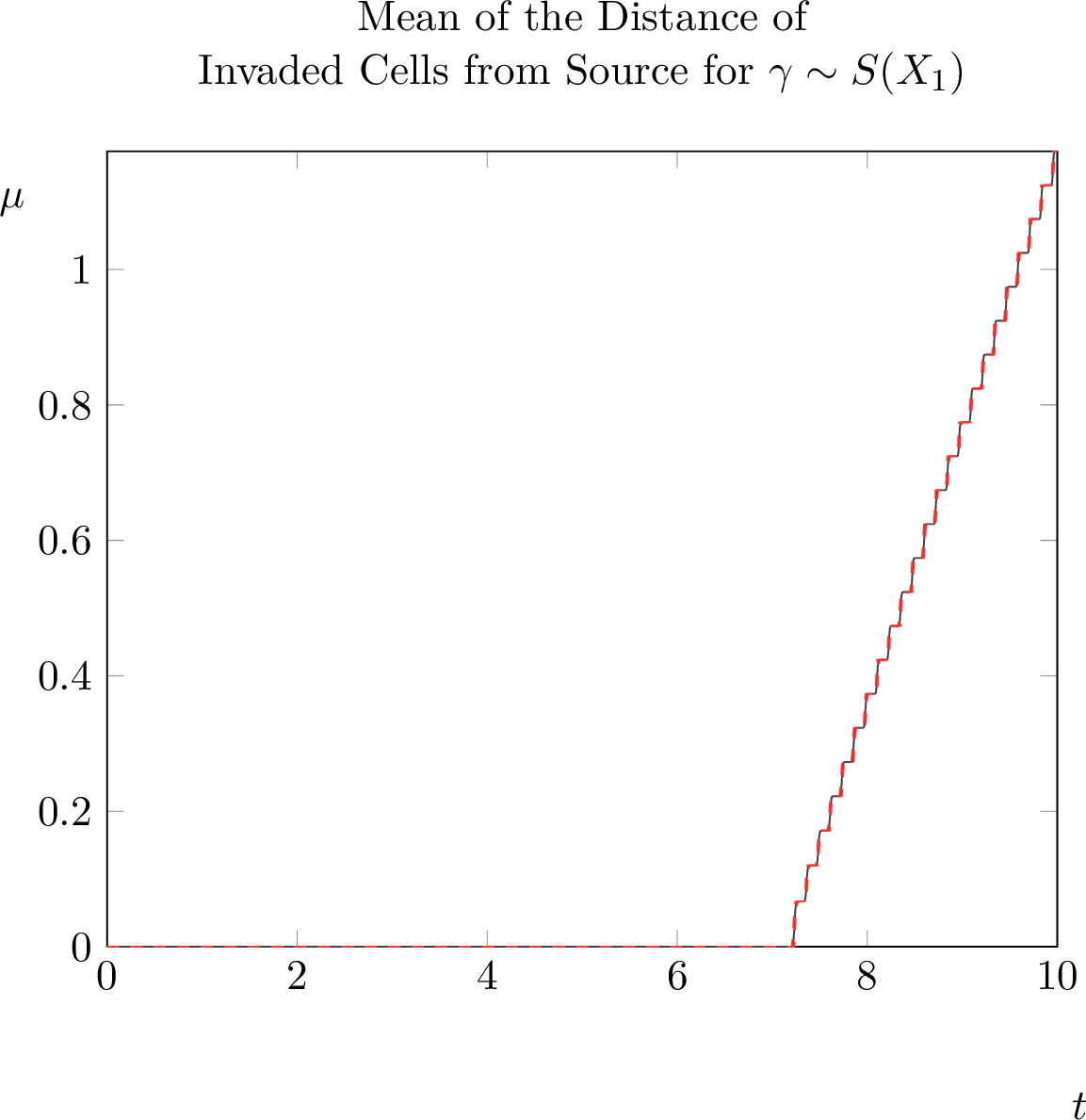

Supplement: S8 Fig — (TIF) [file pone.0300968.s008.tif]

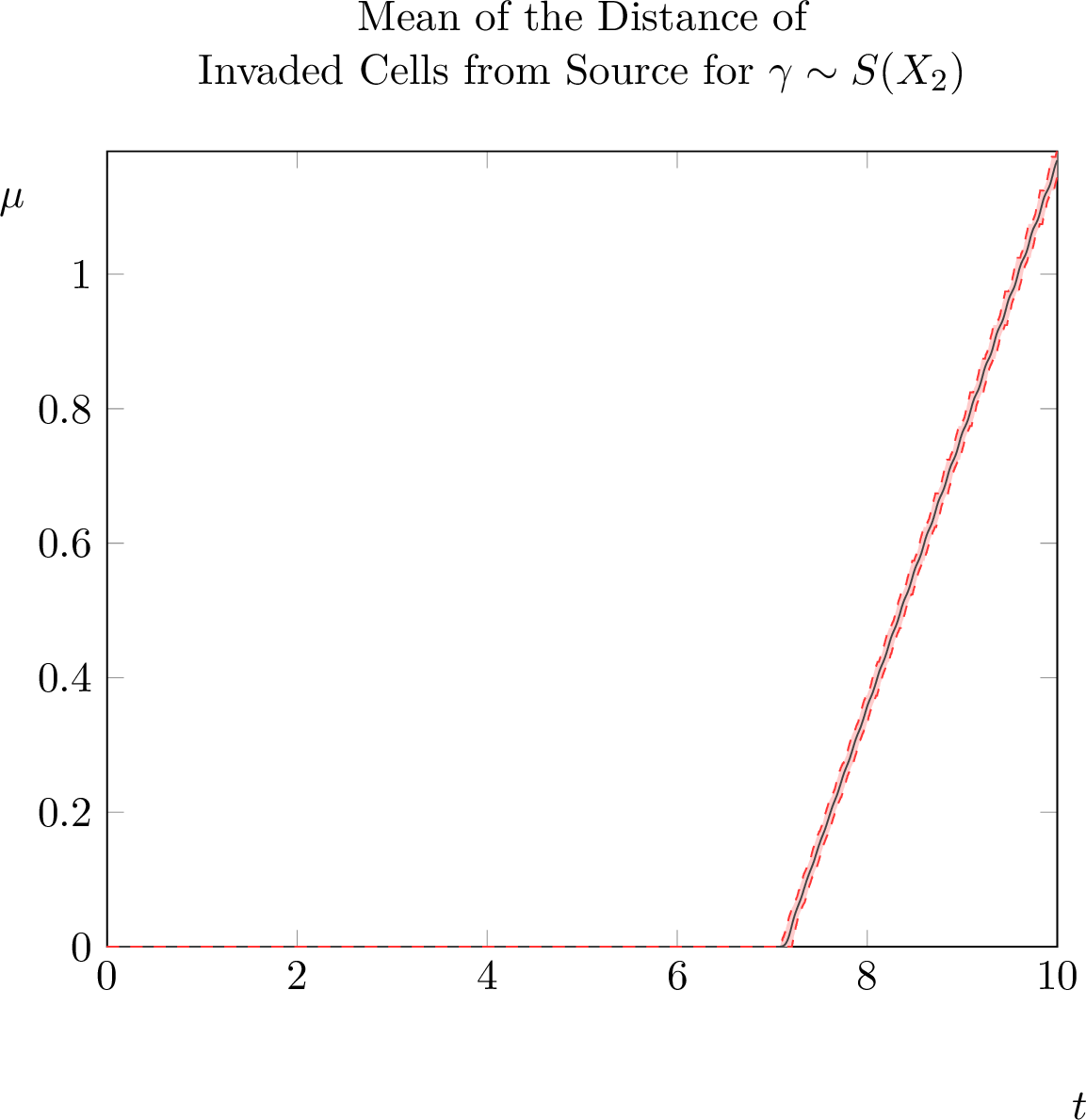

Supplement: S9 Fig — (TIF) [file pone.0300968.s009.tif]

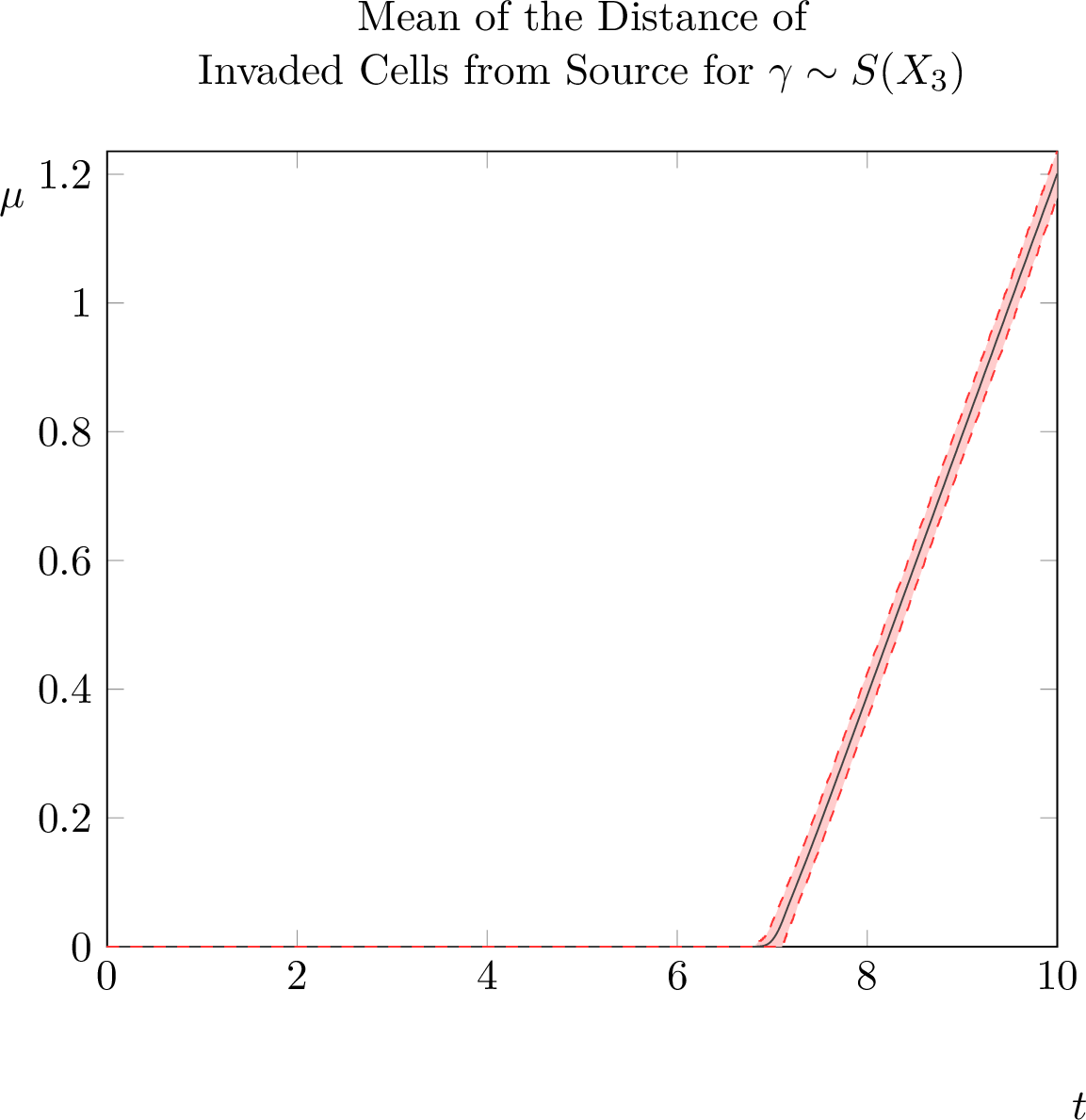

Supplement: S10 Fig — (TIF) [file pone.0300968.s010.tif]
